# A Dual‐Excitation Decoding Strategy Based on NIR Hybrid Nanocomposites for High‐Accuracy Thermal Sensing

**DOI:** 10.1002/advs.202001589

**Published:** 2020-08-25

**Authors:** Shaohua Yu, Jin Xu, Xiaoying Shang, Wei Zheng, Ping Huang, Renfu Li, Datao Tu, Xueyuan Chen

**Affiliations:** ^1^ CAS Key Laboratory of Design and Assembly of Functional Nanostructures State Key Laboratory of Structural Chemistry, and Fujian Key Laboratory of Nanomaterials Fujian Institute of Research on the Structure of Matter Chinese Academy of Sciences Fuzhou Fujian 350002 China; ^2^ University of Chinese Academy of Sciences Beijing 100049 China; ^3^ Fujian Science & Technology Innovation Laboratory for Optoelectronic Information of China Fuzhou Fujian 350108 China

**Keywords:** dual‐excitation decoding, NIR hybrid nanocomposites, photon attenuation, thermal sensing

## Abstract

Optical thermal sensing holds great promise for disease theranostics. However, traditional ratiometric thermometry methods, in which intensity ratio of two nonoverlapping emissions is defined as the thermosensitive parameter, may have a limited accuracy in temperature read‐out due to the deleterious interference from wavelength‐ and temperature‐dependent photon attenuation in tissue. To overcome this limitation, a dual‐excitation decoding strategy based on NIR hybrid nanocomposites comprising self‐assembled quantum dots (QDs) and Nd^3+^ doped fluoride nanocrystals (NCs) is proposed for thermal sensing. Upon excitation at 808 nm, the intensity ratio of two emissions at identical wavelength (1057 nm) from QDs and NCs, respectively, is defined as the thermometric parameter *R*. By employing another 830 nm laser beam following the same optical path as 808 nm laser to exclusively excite QDs, the two overlapping emissions can be easily decoded. The acquired *R* proves to be inert to the detection depth in tissue, with a minimized temperature reading error of ≈2.3 °C at 35 °C (at a depth of ≈1.1 mm), while the traditional thermometry mode based on the nonoverlapping 1025 and 863 nm emissions may exhibit a large error of ≈43.0 °C. The insights provided by this work pave the way toward high‐accuracy deep‐tissue biosensing.

## Graphical abstract

A dual‐excitation decoding strategy, which uses the intensity ratio of two overlapping NIR emissions from self‐assembled QDs and Nd^3+^ doped fluoride NCs in the hybrid nanocomposites, respectively, as the ratiometric thermometric parameter, is proposed for high‐accuracy thermal sensing in vivo.

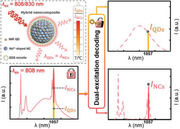

## 1 Introduction

In recent years, in vivo thermal sensing has shown great promise for physiological studies, medical diagnosis, and especially hyperthermia treatment.^[^ ^1^ ^]^ The contactless optical thermometry with both absorption and emission bands lying within the near infrared (NIR) biological window (750–1700 nm) enables deep‐tissue and noninvasive real‐time monitoring of temperature.^[^ ^2^ ^]^ Among them, ratiometric (or self‐referenced) thermometry is well‐established, wherein the intensity ratio of two nonoverlapping emissions with distinct thermal responses is conventionally defined as the thermosensitive parameter.^[^^]^ In this scenario, the temperature read‐out is generally independent of the concentration of thermometers in tissue,^[^^]^ thereby to some extent being beneficial to improve the accuracy of temperature sensing.

Nanothermometers based on lanthanide ions or/and NIR quantum dots (QDs) are among the most promising systems due to their capability of NIR excitation and emission.^[^ ^5^ ^]^ For instance, several examples of Nd^3+^ doped nanocrystals (NCs) by using the intensity ratio of two thermally coupled Stark components of ^4^F_3/2_ multiplet with emission wavelengths close to each other (*I*_1041 nm_/*I*_1062 nm_) have been reported, yet with a much low relative thermal sensitivity (*S*_r_) of less than 0.2% K^−1^ at 27 °C.^[^ ^6^ ^]^ In other examples affording a higher *S*_r_, Carlos et al. reported Nd^3+^/Yb^3+^ codoped NCs relying on the temperature‐dependent intensity ratio of the emission bands of Nd^3+^ at around 1300 nm and Yb^3+^ at ≈1000 nm,^[^ ^2a^ ^]^ and Rodríguez and co‐workers demonstrated a thermal sensing strategy applying the intensity ratio between the thermosensitive emission of QDs at around 1250 nm and thermally inert Nd^3+^ emission at ≈1060 nm.^[^ ^7^ ^]^ Nevertheless, for traditional ratiometric thermometry methods, owing to the difference in photon attenuation coefficient in biological tissue between the two nonoverlapping emissions, the intensity ratio (i.e., the thermometric parameter) varies with the penetration length of light travelling through tissue, which may result in a large error in temperature reading in vivo.^[^ ^8^ ^]^ As pointed out by Liz‐Marzán and co‐workers, an error of tens of degrees in temperature read‐out was expected for deep‐tissue measurements by using typical CaF_2_:Nd^3+^/Y^3+^ nanothermometers.^[^ ^6^ ^]^ Additionally, regarding the reliability of rare‐earth‐doped infrared optical nanothermometers, it was concluded that they are not as reliable as thought.^[^ ^9^ ^]^ That is because the emission spectra of luminescent nanothermometers can be affected by numerous environmental and experimental factors including numerical aperture of the optical elements, the local concentration of nanothermometers, optical length variation, self‐absorption of the luminescence by the nanothermometers themselves, as well as the photon attenuation (absorption and scattering) in ambient medium.^[^ ^9^ ^]^ Specially, with regard to the effect of photon attenuation in medium on the reliability of thermometers, a recent research work discussing the compromised reliability of NIR optical nanothermometers by in vivo spectra distortions has elaborated how the wavelength‐ and temperature‐dependence of the photon attenuation in biological tissue can lead to erroneous measurements of temperature for the traditional optical ratiometric thermometry methods, and has proposed that optical thermometry should include different quality checks to ensure that the presence of the tissue is not leading to erroneous temperature read‐outs.^[^ ^10^ ^]^ Although the published literature has already raised the issue regarding the actual reliability of temperature reading in vivo for the traditional thermometry methods, the issue of how to reliably measure the temperature in vivo persists as a complex puzzle.^[^ ^10^ ^]^ As a result, new optical thermometry strategies in the design of luminescent nanothermometers are crucial to thoroughly solve the problem of unreliability in temperature reading in vivo for the traditional thermometry methods.^[^ ^9^ ^]^

Lights with wavelengths close to each other share homologous photon propagation and attenuation attributes in tissues.^[^ ^8^, ^11^ ^]^ In view of this, Li and co‐workers initiated the time‐resolved ratiometric detection, in which two emission signals with the same working wavelength were used to conduct ratiometric sensing, in order to minimize the deleterious interference from wavelength‐ and temperature‐dependent photon attenuation in tissue.^[^ ^8b,c^ ^]^ Note that, in their ratiometric thermometry method, time‐resolved decoding system luxuriously and complexly equipped with expensive equipments was customized to separate the two overlapping emissions based on their disparate lifetimes.

**Figure 1:**
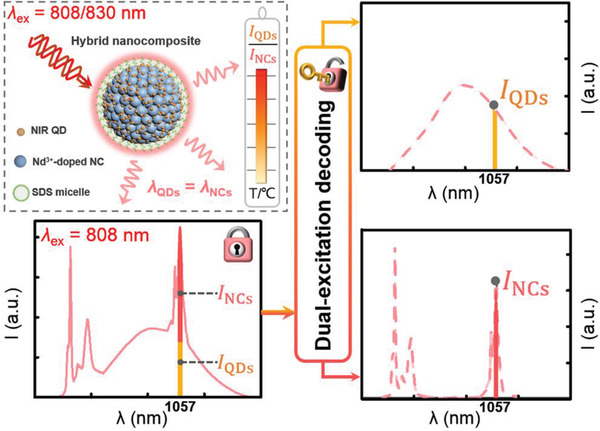
Schematic illustration of the dual‐excitation (808/830 nm) decoding strategy based on hybrid nanocomposites for thermal sensing by using the intensity ratio of two emissions at identical wavelength (1057 nm) as the thermometric parameter.

Herein, a novel dual‐excitation decoding strategy based on hybrid nanocomposites is proposed for thermal sensing. We assembled NIR QDs and Nd^3+^ doped fluoride NCs into hybrid nanocomposites through an emulsion‐based self‐assembly approach to achieve two types of photoluminescence (PL) from QDs and NCs, respectively, with overlapping emissions at 1057 nm upon excitation at 808 nm. By virtue of our dual‐excitation (808/830 nm) decoding strategy, the two overlapping emission signals were separated to acquire their intensity ratio as the thermometric parameter (**Figure** **1**). Furthermore, the capability of this thermometry mode in high‐accuracy thermal sensing was demonstrated by the ex vivo experiments.

## 2 Results and Discussion

**Figure 2:**
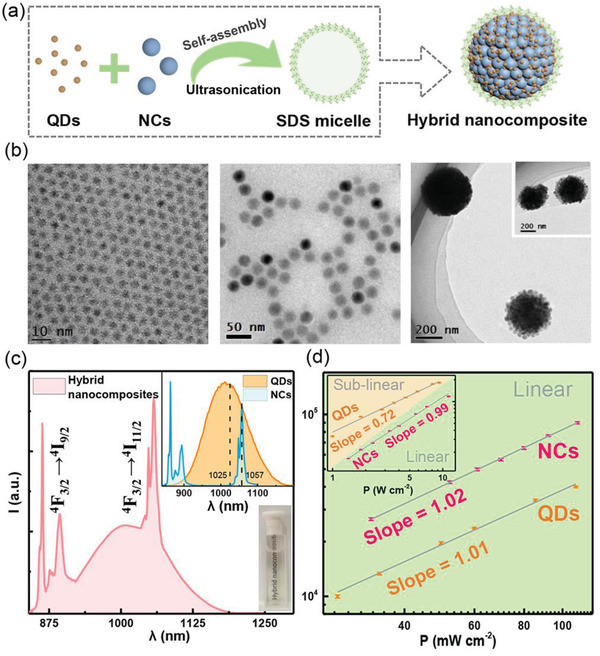
a) Simplified diagram of the synthesis of hybrid nanocomposites. b) TEM images of QDs, NCs and hybrid nanocomposites, respectively. c) PL spectrum of aqueous hybrid nanocomposites dispersion under excitation at 808 nm. The top inset shows PL spectra of QDs and NCs dispersed in cyclohexane, respectively, upon excitation at 808 nm; bottom inset is the photograph of aqueous hybrid nanocomposites dispersion. d) Integrated steady‐state PL intensities of QDs and NCs as a function of *P* of the 808 nm continuous laser, respectively. Each data was presented as average ± standard deviation from three independent measurements.

Aiming at acquiring intense NIR PL signals, the well‐known PbS@CdS@ZnS NIR QDs and hexagonal NaLuF_4_:Gd^3+^/Nd^3+^@NaGdF_4_ fluoride NCs were synthesized and used here as the illustrative examples (Figure S1, Supporting Information).^[^ ^5^, ^12^ ^]^ For the synthesis of hydrophilic hybrid nanocomposites comprising QDs and Nd^3+^ doped fluoride NCs, the as‐prepared hydrophobic QDs and NCs were self‐assembled into sodium dodecyl sulfate (SDS) micellar structures under ultrasonication (**Figure** **2**a).^[^ ^13^ ^]^ Figure 2b shows the transmission electron microscopy (TEM) image of QDs and NCs with average diameters of ≈4 and ≈29 nm, respectively. The representative TEM images of hybrid nanocomposites demonstrated that QDs and NCs were successfully assembled into large microspheres of diameter in the range of 300–400 nm. The prepared hydrophilic hybrid nanocomposites showed a good colloidal stability and strong NIR emission in the region of 850–1200 nm upon 808 nm excitation. The broad excitonic emission band of QDs centered at ≈1010 nm is partially overlapped with the sharp emissions ascribed to the ^4^F_3/2_→^4^I_11/2_/^4^I_9/2_ transitions of Nd^3+^ in fluoride NCs (Figure 2c).^[^ ^14^ ^]^ Due to the remarkable PL thermal quenching effect caused by thermally activated carrier trapping and exciton thermal ionization, etc.,^[^ ^15^ ^]^ QDs were commonly developed as sensitive nanothermometers.^[^ ^16^ ^]^ On the contrary, Nd^3+^ doped fluoride NCs have been more often reported as inner reference unit in ratiometric thermometry, because the ^4^F_3/2_→^4^I_11/2_/^4^I_9/2_ emissions of Nd^3+^ remain virtually unaffected by the temperature changes in the 35–55 °C range (Figures S2 and S3, Supporting Information).^[^ ^7^, ^17^ ^]^

Because of the difference in thermal responses between QDs and Nd^3+^ doped fluoride NCs, the hybrid nanocomposites constructed by these two components can be exploited to create sensitive ratiometric thermometers. The emission of QDs can be used as temperature‐responsive signal (*I*_QDs_), and the ^4^F_3/2_→^4^I_11/2_/^4^I_9/2_ emissions of Nd^3+^ is set as inner reference signal (*I*_NCs_). As for ratiometric thermometry, a prerequisite is that the thermometric parameter *I*_QDs_/*I*_NCs_ should be independent of the excitation power density (*P*).^[^ ^4a^ ^]^ To elaborate this concern, Figure 2d plots the integrated steady‐state PL intensities of QDs and Nd^3+^ doped fluoride NCs as a function of *P* of the 808 nm laser, respectively. It can be clearly seen that the integrated PL intensity of QDs was linearly dependent on *P* (*I*(PL) ∝ *P*) in the low power regime of ≈20–110 mW cm^−2^, owing to the dominant radiative recombination of free carriers.^[^ ^18^ ^]^ Unexpectedly, at relatively higher *P* regime (e.g., *P* > 1 W cm^−2^), the power law changed from linear to sub‐linear (*I*(PL) ∝ *P* ^0.72^) (inset of Figure 2d), due possibly to the enhanced Auger processes.^[^ ^19^ ^]^ Additionally, the PL intensity of Nd^3+^ doped fluoride NCs was linearly dependent on *P*, even in the high power regime (Figure 2d).^[^ ^17a^ ^]^ Therefore, in the following experiments, the power density of NIR‐laser irradiation on the hybrid nanocomposites was set in the range of ≈20–110 mW cm^−2^. In this way, it is guaranteed that both PL signals of QDs and NCs increased linearly with *P* (i.e., *I*_QDs_/*I*_NCs_ was independent of *P*), and the PL signal of QDs or NCs after travelling through a several mm‐thick tissue were sufficiently detectable over the noise of the photon detector.

**Figure 3:**
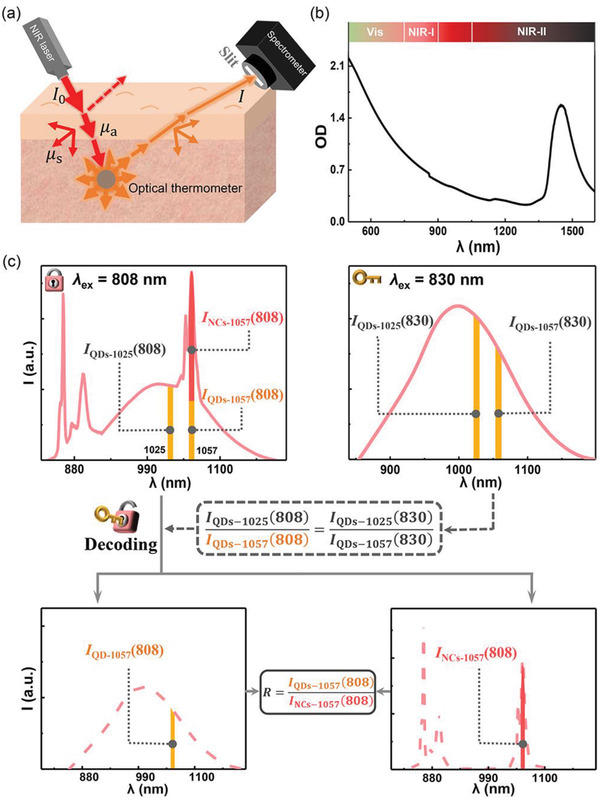
a) Sketch map of the setup for optical thermometry in vivo (*μ*_s_ and *μ*_a_ are scattering and absorption coefficients, respectively). b) Extinction spectrum of tissue phantom. The tissue phantom consists of 1% Intralipid in water to simulate the photon attenuation in pork tissue.^[^ ^22^ ^]^ c) Schematic diagram of the dual‐excitation (808/830 nm) decoding strategy for the acquisition of thermometric parameter *R* (*I*_QDs‐1057_(808)/*I*_NCs‐1057_(808)).

For traditional thermometry methods, to acquire a high *S*_r_ and/or to facilitate the discrimination of detecting signals, the intensity ratio of two nonoverlapping emissions with distinct thermal responses is defined as the ratiometric thermometric parameter.^[^ ^7^, ^20^ ^]^ However, lights with different wavelengths differ in photon attenuation coefficient in tissue (**Figure** **3**a,b). Thus, the larger the difference in wavelength is, the bigger the error in temperature reading becomes.^[^ ^8b^ ^]^ To address this issue, herein, based on the aforementioned hybrid nanocomposites, the overlapping emission signals at 1057 nm (emitted from QDs and NCs, respectively, under excitation at 808 nm) will be separated to acquire their intensity ratio as the thermometric parameter (Figure 3c). It is worth noting that Nd^3+^ doped NCs show an absorption gap between the ^4^I_9/2_→^4^F_3/2_ and ^4^I_9/2_→^4^F_5/2_/^2^H_9/2_ transitions of Nd^3+^ (in the range of ≈823–853 nm), contrary to the continuum intraband absorption of QDs ranging from ultraviolet to 1200 nm (Figure S4, Supporting Information).^[^ ^21^ ^]^ As a result, upon 808 nm excitation, broad excitonic emission band of QDs partially overlapped with the sharp emission lines of Nd^3+^ was observed in the hybrid nanocomposites (Figure 2c, upper left of Figure 3c). By contrast, only the broad excitonic emission of QDs was produced under excitation at 830 nm (upper right of Figure 3c). Consequently, using another 830 nm laser beam to solely excite QDs in hybrid nanocomposites may be a key capable of unlocking the overlapping emission signals at 1057 nm under 808 nm excitation (Figure 3c).

Prior to discussing how to separate the overlapping emission signals in practical thermal sensing process, we will firstly prove that the intensity ratio of emission at 1025 nm to that at 1057 nm for QDs (*I*_QDs‐1025_/*I*_QDs‐1057_) in the hybrid nanocomposites under 808 nm excitation equals the case of 830 nm excitation. In consideration of the attenuation (scattering and absorption) effect of light travelling through tissue (Figure 3a), *I*_QDs‐1025_/*I*_QDs‐1057_ is given by

(1) $$\begin{array}{c} \begin{array}{ccc} \frac{I_{\mathrm{QDs}-1025}}{I_{\mathrm{QDs}-1057}} & = & \frac{\sum I_{i}\cdot A_{i}\cdot \eta_{1025}\cdot \varphi_{i}\left(\mu_{s(1025)},\mu_{a(1025)},g_{1025},{\vec{r}}_{i}\right)}{\sum I_{i}\cdot A_{i}\cdot \eta_{1057}\cdot \varphi_{i}\left(\mu_{s(1057)},\mu_{a(1057)},g_{1057},{\vec{r}}_{i}\right)}\\ & = & C\cdot \frac{\sum \left(\frac{I_{i}}{I_{\max}}\right)\cdot \left(\frac{A_{i}}{A_{\max}}\right)\varphi_{i}\left(\mu_{s(1025)},\mu_{a(1025)},g_{1025},{\vec{r}}_{i}\right)}{\sum \left(\frac{I_{i}}{I_{\max}}\right)\cdot \left(\frac{A_{i}}{A_{\max}}\right)\cdot \varphi_{i}\left(\mu_{s(1057)},\mu_{a(1057)},g_{1057},{\vec{r}}_{i}\right)} \end{array} \end{array}$$

where *I*_i_ represents the laser beam intensity reaching on the surface of hybrid nanocomposite particle i in tissue, and *I*_i_/*I*_max_ means that *I*_i_ is normalized to the maximum intensity *I*_max_; *A*_i_ is the dimensionless absorption strength of QDs in hybrid nanocomposite particle i, and *A*_i_/*A*_max_ indicates that *A*_i_ is normalized to the maximum absorption strength *A*_max_; *η*_1025_ (*η*_1057_) is the absolute quantum yield of emission of QDs at 1025 (1057) nm; *φ*_i_ is the signal response function of the experimental system for the emission from particle i; and *C* is a constant (*C* = *η*_1025_/*η*_1057_).

Herein, under the diffusion approximation (Figure S5, Supporting Information),^[^ ^11^ ^]^ *φ*_i_ should include contributions of the scattering coefficient *μ*_s_, absorption coefficient *μ*_a_, scattering anisotropic factor *g*, as well as position vector ${\vec{r}}_{i}$ between particle i and the slit of spectrometer of the experimental system. As indicated by the dimensionless parameter *I*_i_/*I*_max_ in Equation (1), *I*_QDs‐1025_/*I*_QDs‐1057_ depends on the intensity distribution law of laser beam in tissue. Considering that the attenuation of laser beam is predominantly caused by tissue, the minute contribution to photon attenuation of laser beam from light scattering/absorption by hybrid nanocomposites (with a low concentration in the order of mg mL^−1^) can be rationally neglected.^[^ ^11^ ^]^ Thus, the intensity distribution laws in tissue (*I*_i_/*I*_max_) for 808 and 830 nm laser beams with close wavelengths are nearly identical to each other.^[^ ^8^, ^23^ ^]^ In the meantime, if the excitations of hybrid nanocomposites by 808 and 830 nm lasers and their corresponding emission signal collections are conducted in the identical experimental optical system, the parameters *A*_i_/*A*_max_ and *φ*_i_ in Equation (1) should be the same in the cases of 808 and 830 nm excitations. Under such circumstances, *I*_QDs‐1025_/*I*_QDs‐1057_ under 808 nm excitation equals the case of 830 nm excitation, *i.e*., *I*_QDs‐1025_(808)/*I*_QDs‐1057_(808) = *I*_QDs‐1025_(830)/*I*_QDs‐1057_(830).

Based on the above analysis, the two overlapping emission signals at 1057 nm emitted from hybrid nanocomposites under 808 nm excitation can be easily separated to acquire the thermometric parameter *I*_QDs‐1057_(808)/*I*_NCs‐1057_(808) through a dual‐excitation (808/830 nm) decoding strategy. To be specific, the emission intensity of QDs at 1057 nm *I*_QDs‐1057_(808) = *I*_QDs‐1057_(830)/*I*_QDs‐1025_(830) × *I*_QDs‐1025_(808), and the emission intensity of NCs at 1057 nm *I*_NCs‐1057_(808) = *I*_total_ – *I*_QDs‐1057_(808), where *I*_total_ is total intensity of the overlapping NIR emissions at 1057 nm (Figure 3c). Accordingly, the thermometric parameter *I*_QDs‐1057_(808)/*I*_NCs‐1057_(808) (hereafter *R*) can thus be calculated. Moreover, it proved that the theoretical value of *R* at a fixed ambient temperature is substantially proportional to the average molar ratio of QDs to NCs (*n*_QDs_/*n*_NCs_) in the hybrid nanocomposites (Equations (2) and (3)) in the Experimental Section). In other words, theoretically, *R* is independent of the depth of thermometers in tissue. In practical thermal sensing in vivo, it is conceivable that the excitation lights with different wavelengths (i.e., 808 and 830 nm) may cause a temperature reading error. Actually, the possible temperature reading error caused by the excitation lights with different wavelengths originates from the difference in the intensity distribution laws in tissue between 808 and 830 nm laser beams, as depicted by the dimensionless parameter *I*_i_/*I*_max_ in Equation (1). On the other hand, due to the steep photon attenuation of emission light with an increase in the light propagation distance in tissue, the light‐emitting zone, whose emission signals are effectively collected by the grating spectrometer, is constrained in a minute volume in tissue. As such, it is rational to deduce that, within the minute volume in tissue, the small difference in the intensity distribution laws between the 808 and 830 nm laser beams has little influence on the accuracy of temperature reading in vivo.

**Figure 4:**
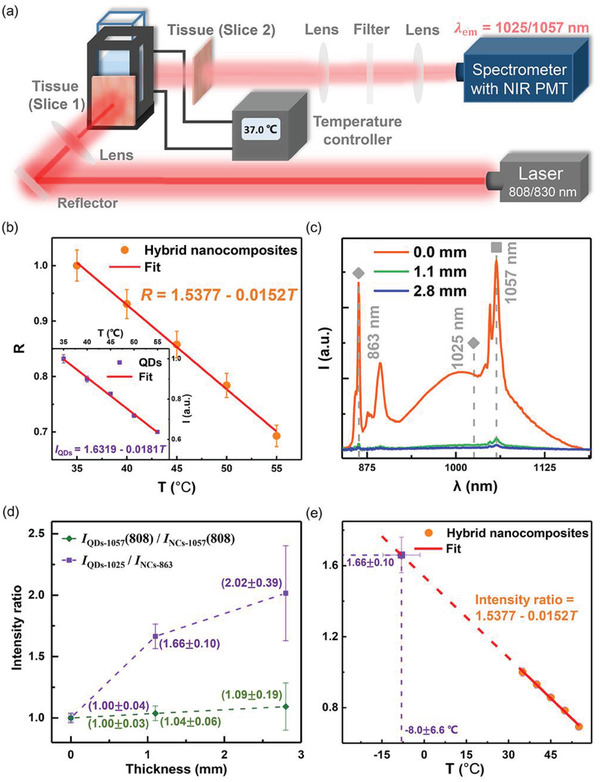
a) Schematic diagram of experimental optical system for the ex vivo experiments. b) *R* of the hybrid nanocomposites thermometers and PL peak intensity of QDs (inset) as a function of temperature, respectively, and their corresponding linear regression equations. The *R* was normalized to the value at 35.0 °C. Each data was presented as average ± standard deviation calculated through Equation (4b) in the Experimental Section. The data in inset were presented as average ± standard deviation from three independent measurements. c) PL spectra of the hybrid nanocomposites versus thickness of Slice 2 pork tissue. d) *R* and the intensity ratio between two nonoverlapping emissions at 1025 and 863 nm (*I*_QDs‐1025_/*I*_NCs‐863_) versus thickness of Slice 2 pork tissue, respectively, at the ambient temperature of 35.0 °C. The *R* or *I*_QDs‐1025_/*I*_NCs‐863_ was normalized to the value corresponding to the case of thickness of 0 mm. Data were presented as average ± standard deviation calculated from three independently measured spectra of the hybrid nanocomposites. e) Temperature read‐out by using the linear equation derived from the regression analysis of *R* versus *T* in (b).

The applicability of this dual‐excitation decoding strategy in thermal sensing was then demonstrated through the experimental scheme as depicted in **Figure** **4**a. An aqueous solution of hybrid nanocomposites thermometers was placed in a cuvette equipped with an external temperature controller, and the excitation sources (808 and 830 nm laser beams) were along the same optical path. The thermometric parameter *R* (i.e., *I*_QDs‐1057_(808)/*I*_NCs‐1057_(808)) was acquired through the aforementioned protocol. The *R* value decreased rapidly to show a linear tendency with an increase in temperature (Figure 4b). Similarly, the PL intensity of QDs upon 830 nm excitation also exhibited a linear decrease with the temperature rise (inset of Figure 4b).^[^ ^7^ ^]^ The hybrid nanocomposites thermometers exhibited good stability and reversibility (Figures S6 and S7, Supporting Information), and the *S*_r_ (*S*_r_ = (1/*R*) · (d*R*/d*T*)) and thermal resolution were ≈1.5% K^−1^ and ≈1.8 °C at 35.0 °C, respectively, as calculated in Equations (4a) and (4b) in the Experimental Section. Note that the *S*_r_ of the hybrid nanocomposites thermometers was in close proximity to that of the QDs themselves (≈1.8% K^−1^ at 35.0 °C; for QDs, *S*_r_ = (1/*I*) · (d*I*/d*T*)) (Figure 4b). Such a similarity in *S*_r_ is understandable, because the hybrid nanocomposites thermometers with the Nd^3+^ emission as inner reference signal completely succeeded the thermal sensitivity of QDs themselves. Besides the thermometric parameter *R*, the intensity ratio of two emissions at 1025 and 863 nm exclusively belonging to QDs and NCs, respectively (Figure 4c), can also be defined as a temperature‐responsive parameter (i.e., *I*_QDs‐1025_/*I*_NCs‐863_). Actually, such a mode was often adopted in traditional thermometry, for the sake of easy discrimination of detecting signals.^[^ ^7^, ^17^, ^20^ ^]^

To verify the superiority of the dual‐excitation decoding strategy in thermal sensing in vivo over traditional thermometry mode, the thermometric parameters *R* and *I*_QDs‐1025_/*I*_NCs‐863_ were compared in the accuracy of temperature reading. The in vivo conditions were simulated by adding a slice of pork tissue (marked as Slice 1) in front of the cuvette, with another slice of pork tissue (Slice 2) placed at 90° with respect to the illumination axis (Figure 4a and Figure S8, Supporting Information). Here, the thickness of Slice 1 was fixed at ≈0.6 mm, and that of Slice 2 was kept variable. With an increase in the thickness of Slice 2 pork tissue, the PL intensity of QDs (or NCs) and the signal‐to‐background ratio (SBR) of emission signal both diminished steeply (Figure 4c). The *R* and *I*_QDs‐1025_/*I*_NCs‐863_ (calculated from the spectrum in Figure 4c) as a function of the thickness of Slice 2 pork tissue were plotted in Figure 4d, respectively. A slight change in *R* with the thickness was observed. Ideally, *R* should be independent of the tissue thickness (Equations (2) and (3) in the Experimental Section). In the actual situation, since *R* is defined as the intensity ratio of two emission signals, it is conceivable that the calculated *R* value is susceptible to the decreasing SBR of emission signal. Such an interference to *R* was evidenced by the considerable increase in the uncertainty of *R* value with increasing tissue thickness (Figure 4d and **Table** **1**). Accordingly, the perceptible change in *R* with the tissue thickness was primarily caused by the interferences from the instrumental noise. In sharp contrast to the case of *R*, the *I*_QDs‐1025_/*I*_NCs‐863_ increased markedly with the thickness (Figure 4d). Note that the absolute variation from 1.00 (i.e., |*I*_QDs‐1025_/*I*_NCs‐863_ – 1.00|) was several times larger than the uncertainty of *I*_QDs‐1025_/*I*_NCs‐863_ value for each tissue thickness (1.1, 2.8 mm) (Figure 4d). Thus, it is rational to deduce that the remarkable change in *I*_QDs‐1025_/*I*_NCs‐863_ with the tissue thickness was mainly caused by the difference in photon attenuation coefficient between the 1025 and 863 nm emissions (Figure 3b). At the ambient temperature of 35.0 °C, the calculated temperatures by using *R* and *I*_QDs‐1025_/*I*_NCs‐863_ as the thermometric parameters were ≈32.7 ± 4.0 and ≈−8.0 ± 6.6 °C, respectively, corresponding to the simulated condition that the hybrid nanocomposites thermometers were at the depth of 1.1 mm in pork tissue (Figure 4e and Table 1). Clearly, the error in temperature read‐out by using our dual‐excitation decoding strategy was ≈2.3 °C at 35.0 °C, close to the thermal resolution of thermometers (≈1.8 °C), while for traditional thermometry mode it was as large as ≈43.0 °C. **Table** **2** listed representative optical ratiometric thermometry methods with emission bands lying within the NIR biological window. For these thermometry methods commonly used in bioapplications, intensity ratio of two nonoverlapping emissions is defined as the thermometric parameter in consideration of the easy discrimination of detecting signals. However, such methods are confronted by the problem of unreliability in deep‐tissue temperature reading, which substantially stems from the difference in photon attenuation coefficient in biological tissue between the two nonoverlapping emissions.

**Table 1:** Comparison of thermal sensing accuracy between our dual‐excitation decoding strategy and traditional thermometry mode (the ambient temperature is 35.0 °C)

	Dual‐excitation decoding strategy		Traditional thermometry mode	
Tissue thickness [mm]	*R*	Calculated temperature [°C]	*I*_QDs‐1025_/*I*_NCs‐863_	Calculated temperature [°C]
0	1.00 ± 0.03	35.0 ± 2.0	1.00 ± 0.04	35.0 ± 2.6
**1.1**	**1.04 ± 0.06**	**32.7 ± 4.0**	**1.66 ± 0.10**	**−8.0 ± 6.6**
2.8	1.09 ± 0.19	29.5 ± 12.5	2.02 ± 0.39	−31.7 ± 25.7

**Table 2:** Representative optical ratiometric thermometry methods with emission bands lying within the NIR biological window

		Ratio (*I*_em1_/*I*_em2_)				
Material	Emission bands	*λ*_em1_ [nm]	*λ*_em1_ [nm]	Anti‐interference^a)^	*S*_r_ [% K^−1^]	Ref.
NaYF_4_:Yb^3+^/Tm^3+^@CaF_2_	Tm^3+^:^3^H_4_→^3^H_6_ (Stark^b)^)	802	820	No	0.43 (313 K)	^[^ ^24^ ^]^
NaNbO_3_:Tm^3+^	^3^H_4_→^3^H_6_ (Stark)	797	807	No	0.80 (303 K)	^[^ ^25^ ^]^
LiLaNdYbP_4_O_12_	Nd^3+^:^4^F_3/2_→^4^I_9/2_ , Yb^3+^:^2^F_5/2_→^2^F_7/2_	870	980	No	0.40 (330 K)	^[^ ^26^ ^]^
NaGdF_4_:Yb^3+^/Ho^3+^/Er^3+^@NaGdF_4_: Yb^3+^@NaGdF_4_:Yb^3+^/Nd^3+^	Er^3+^:^4^I_13/2_→^4^I_15/2_, Ho^3+^:^5^I_6_→^5^I_8_, Nd^3+^:^4^F_3/2_→^4^I_13/2_	1180 or 1550	1340	No	1.10 (273 K)	^[^ ^27^ ^]^
LaF_3_:Nd^3+^	^4^F_3/2_→^4^I_9/2_ (Stark)	885	865	No	0.26	^[^ ^28^ ^]^
Y_3_Al_5_O_12_:Nd^3+^	^4^F_3/2_→^4^I_9/2_ (Stark)	938	945	No	0.15	^[^ ^29^ ^]^
LaF_3_:Nd^3+^	^4^F_3/2_→^4^I_9/2_ (Stark)	863	886	No	0.20	^[^ ^30^ ^]^
CaF_2_:Y^3+^/Nd^3+^	^4^F_3/2_→^4^I_11/2_ (Stark)	1041	1062	No	0.16 (300 K)	^[^ ^6^ ^]^
LaF_3_:Nd^3+^@ LaF_3_:Yb^3+^	Nd^3+^:^4^F_3/2_→^4^I_13/2_, Yb^3+^:^2^F_5/2_→^2^F_7/2_	1300	1000	No	0.41 (283 K)	^[^ ^2a^ ^]^
NaYF_4_:Nd^3+^	^4^F_3/2_→^4^I_9/2_ (Stark)	863	870	No	0.12 (273 K)	^[^ ^31^ ^]^
TTA‐NaYF_4_: Nd^3+^ (TTA: triplet–triplet annihilation)	TTA:upconversion emission, Nd^3+^:^4^F_3/2_→^4^I_11/2_	540	1060	No	7.10	^[^ ^17a^ ^]^
PbS@CdS@ZnS + NaGdF_4_:Nd^3+^	PbS/CdS/ZnS:excitonic emission, Nd^3+^:^4^ F_3/2_→^4^I_11/2_	1250	1060	No	2.50 (293 K)	^[^ ^7^ ^]^
NaYF_4_:Yb^3+^/Ho^3+^/Er^3+^	Ho^3+^:^5^I_6_→^5^I_8_, Er^3+^:^4^I_13/2_→^4^I_15/2_	1150	1550	No	–	^[^ ^20a^ ^]^
PbS@CdS@ZnS + NaLuF_4_:Gd^3+^/Nd^3+^@NaGdF_4_	PbS/CdS/ZnS:excitonic emission, Nd^3+^:^4^ F_3/2_→^4^I_11/2_	1057	1057	Yes	1.50 (308 K)	This work

a) Anti‐interference means that the deleterious interference from wavelength‐ and temperature‐dependent photon attenuation in tissue can be avoidable;

b) Thermally coupled Stark components of multiplet.

In order to further demonstrate the potential application of our dual‐excitation decoding strategy in practical thermal sensing in vivo, ex vivo experiment consisting of direct intra‐tissue injection of hybrid nanocomposites thermometers was proceeded to determine the local temperature in animal tissue (pork). A volume of 80 µL of an aqueous solution of hybrid nanocomposites (5 mg mL^−1^) was injected at a depth of ≈1 mm into the pork tissue that was placed on a heating device dynamically set at different temperatures in the range of 30–52 °C. The calculated local temperatures in tissue by using the linear equation derived from the regression analysis of *R* versus *T* in Figure 4b were shown in Figure S9 (Supporting Information) and compared with the setting temperatures of heating device. The temperature values acquired from the dual‐excitation decoding strategy presented a very good agreement with the setting temperatures, indicating the reliability of the proposed thermometry strategy in a real detection situation in vivo.

## 3 Conclusion

In summary, we have proposed a prototypical dual‐excitation decoding strategy based on hybrid nanocomposites for thermal sensing, wherein the intensity ratio of two emissions at identical wavelength was defined as the thermometric parameter to avoid deleterious interference from wavelength‐ and temperature‐dependent photon attenuation in tissue. We elaborately designed the hybrid nanocomposites composed of self‐assembled NIR QDs and Nd^3+^ doped inorganic NCs to acquire the overlapping emissions at 1057 nm ascribed to QDs and NCs, respectively, under 808 nm excitation. Upon excitation with another 830 nm laser beam following the same optical path as 808 nm laser, the overlapping emission signals can be decoded to acquire the thermometric parameter. Furthermore, we verified in the proof‐of‐concept ex vivo experiments that the proposed dual‐excitation decoding strategy was capable of achieving high‐accuracy temperature reading with a small error of ≈2.3 °C at 35.0 °C for the detection depth of ≈1.1 mm in pork tissue (*S*_r_ ≈ 1.5% K^−1^ and thermal resolution ≈ 1.8 °C at 35 °C). By contrast, for traditional thermometry mode by using the intensity ratio of two nonoverlapping emissions with distinct thermal responses as the thermosensitive parameter, a large error up to tens of degrees may occur in deep‐tissue measurements. These findings open up a new avenue for high‐accuracy thermal sensing in vivo and provide a general dual‐excitation decoding strategy that is promising for a variety of deep‐tissue ratiometric biodetections.

## 4 Experimental Section

##### Chemicals and Materials

Ln_2_O_3_ (Ln = Lu, Gd, Nd,) (99.99%), cyclohexane, toluene, acetone, methanol, ethanol, zinc oxide (ZnO, analytical reagent grade) and sodium dodecyl sulfate (SDS) were purchased from Sinopharm Chemical Reagent Co., China. Oleic acid (OA, 90%), oleylamine (OM, 90%), 1‐octadecence (ODE, 90%) and bis(trimethylsilyl) sulfide ((TMS)_2_S, synthesis grade) were purchased from Sigma‐Aldrich (China). Trifluoroacetic acid, lead oxide (PbO, 99.99%) and cadmium oxide (CdO, 99.99%) were purchased from Aladdin (Shanghai, China). All the chemical reagents were used as received without further purification.

##### Synthesis of NaLuF_4_:Gd^3+^/Nd^3+^@NaGdF_4_ Nanocrystals (NCs)

NaLuF_4_:Gd^3+^/Nd^3+^ NCs were synthesized via a modified thermal decomposition process.^[^ ^12c^ ^]^ Ln(CF_3_COO)_3_ (0.5 mmol, 62% mol of Lu, 33% mol of Gd, 5% mol of Nd,) and Na(CF_3_COO) (0.75 mmol) were mixed with OA (5.9356 g), OM (2.9627 g), and ODE (3.0197 g) in a 100 mL flask at room temperature (RT). The obtained mixture was heated at 120 °C under a N_2_ flow with constant stirring for 30 min to form a clear solution and remove the residual water and oxygen. Subsequently, the solution was heated to 310 °C and stabilized for 60 min, and then cooled to RT naturally. The resulting NaLuF_4_:Gd^3+^/Nd^3+^ NCs were precipitated by addition of ethanol, collected via centrifugation, washed with ethanol for two times, and redispersed in cyclohexane for the next step of synthesis. Afterward, the core–shell structured NaLuF_4_:Gd^3+^/Nd^3+^@NaGdF_4_ NCs were fabricated by using the seed‐mediated method. Half of previously synthesized NaLuF_4_:Gd^3+^/Nd^3+^ NCs were added to a 100 mL flask with OA (6.2954 g) and ODE (5.5626 g). The solution was stirred at 90 °C in N_2_ atmosphere to remove cyclohexane. The reaction mixture was then cooled to RT. Thereafter, Gd(CF_3_COO)_3_ (0.425 mmol) and Na(CF_3_COO) (0.425 mmol) were added. The mixture was heated at 120 °C for 15 min under magnetic stirring in N_2_ atmosphere to dissolve the trifluoroacetate precursors and remove the residual water and oxygen. Subsequently, the resulting transparent solution was heated to 300 °C with vigorous stirring for 60 min, and then cooled to RT naturally. The resulting NaLuF_4_:Gd^3+^/Nd^3+^@NaGdF_4_ NCs were precipitated by addition of ethanol, collected via centrifugation, washed with ethanol for two times, and redispersed in cyclohexane.

##### Synthesis of PbS@CdS@ZnS Quantum Dots

In a typical hot injection method,^[^ ^12a^ ^]^ PbO (0.4 mmol) was added to a 50 mL flask containing OA (1.335 g) and ODE (1.9725 g) at RT. The obtained mixture was heated at 120 °C for 30 min under magnetic stirring in N_2_ atmosphere to dissolve the lead precursors and remove the residual water and oxygen. The solution was then cooled to 100 °C and stabilized for 10 min, followed by rapid injection of 2 mL of sulfur precursor (0.2 mmol (TMS)_2_S in 2 mL of ODE ). After 3 min, the reaction mixture was cooled down to RT by ice‐water bath. The resulting PbS QDs were precipitated by addition of acetone, collected via centrifugation, washed with acetone for several times, and re‐dispersed in cyclohexane. Afterward, CdS shell was formed by a cation exchange method. CdO (0.25 g) was dissolved in OA (2.6981 g) and ODE (7.9466 g) by heating. The obtained solution was then cooled down to 60 °C, followed by injection of PbS dispersion with syringe. Subsequently, the reaction solution was heat to 100 °C and maintained for 1 h. After the thin CdS shell formation, the reaction flask was cooled to RT. The resulting PbS@CdS QDs were precipitated by addition of mixture of methanol, acetone, and toluene, and then purified by centrifugation. The obtained PbS@CdS QDs were added into ODE and heated to 240 °C under N_2_ atmosphere, followed by injection of zinc precursor (ZnO dissolved in OA and ODE, 0.5 m) for further growth of ZnS shell. After 5 min, sulfur precursor solution (sulfur dissolved in ODE, 0.5 m) was injected and the temperature was maintained for an additional 5 min. The sequential injections of zinc and sulfur precursors were repeated for two times. After that, the reaction solution was cooled to RT and the final product was precipitated by addition of mixture of methanol, acetone, and toluene, and then purified by centrifugation. The precipitation was washed with methanol several times and re‐dispersed in cyclohexane.

##### Synthesis of Hybrid Nanocomposites

The hybrid nanocomposites were synthesized via a emulsion‐based self‐assembly method.^[^ ^13^ ^]^ In order to prepare the water‐dispersed hybrid nanocomposites from oil‐dispersed NCs, SDS (15 mg) was added to distilled water (10 mL). A cyclohexane solution containing PbS@CdS@ZnS QDs and NaLuF_4_:Gd^3+^/Nd^3+^@NaGdF_4_ NCs was added dropwise to the SDS aqueous solution under ultrasonication. Subsequently, the resulting solution was heated to 70 °C with constant stirring for 4 h to remove the residual cyclohexane. After the reaction solution was cooled to RT, the products were purified by repeated redispersion and centrifugation, and were re‐dispersed in water.

##### Experimental Optical System for Ex Vivo Experiments

A quartz cuvette containing aqueous hybrid nanocomposites dispersion (2.5 mL, ≈5 mg mL^−1^) was placed in a sample holder equipped with an external temperature controller (APCT‐2, Automatic Science Instrument CO., LTD.). A nonmode‐locked Ti: sapphire oscillator (710–920 nm, Maitai, Spectra Physics) (i.e., laser output is continuous) was utilized to generate 808 and 830 nm laser (FWHM ≈4 nm) to insure that the two lasers share the same optical path. The photoluminescence (PL) was collected by the lens and was recorded by a FLS 980 spectrometer (Edinburgh) with NIR detector (InGaAs PMT). For the signal acquisitions, the PL intensity at 1025/1057 nm was monitored with a slit of 3 nm; the PL full spectrum was recorded with a slit of 0.7 nm. The recording process of PL intensity at 1025/1057 nm was set to be less than 1 s, fast enough to avoid any side‐effect due to laser heating.

##### Structural and Optical Characterization

Powder X‐ray diffraction patterns (XRD) of the samples were collected with an X‐ray diffractometer (MiniFlex2, Rigaku) with Cu K*α*1 radiation (*λ* = 0.154187 nm). Transmission electron microscopy (TEM) measurements were performed by using a FEI Tecnai F20 TEM. Absorption measurements were collected by UV–vis–NIR spectrophotometer (Lambda 950, PerkinElmer). PL excitation and emission spectra were acquired on the FLS980 spectrometer equipped with continuous xenon (450 W). The absolute PL quantum yields (QYs) of QDs and NCs were measured by employing a standard barium sulfate coated integrating sphere (150 mm in diameter, Edinburgh) as the sample chamber that was mounted on the FLS980 spectrometer with the entry and output port of the sphere located in 90° geometry from each other in the plane of the spectrometer. Three independent measurements were carried out to yield the average value and standard deviation. A standard tungsten lamp was used to correct the optical response of the instrument. All the spectral data were recorded at room temperature and corrected for the responses of both the spectrometer and the integrating sphere. Thermal stage in a closed‐cycle liquid helium cryostat (CS202PE‐DMX‐1AL, 10–325 K) was used as the heating device for the ex vivo experiment consisting of direct intra‐tissue injection of hybrid nanocomposites thermometers.

##### Theoretical Value of Thermometric Parameter *I*_QDs‐1057_(808)/*I*_NCs‐1057_(808)

Taking into accounts the scattering and absorption of light travelling in tissue, the intensity ratio between emissions of QDs and NCs in the hybrid nanocomposites at 1057 nm under 808 nm excitation is determined by

(2) $$\begin{array}{ccc} \frac{I_{\mathrm{QDs}-1057}\left(808\right)}{I_{\mathrm{NCs}-1057}\left(808\right)} & = & \frac{\eta_{1057}}{\eta_{1057}^{\prime }}\frac{\sum I_{i}\cdot {A_{i}}_{\left(808\right)}\cdot \varphi_{i}\left(\mu_{s(1057)},\mu_{a(1057)},g_{1057},{\vec{r}}_{i}\right)}{\sum I_{i}\cdot A_{i(808)}^{\prime }\cdot \varphi_{i}\left(\mu_{s(1057)},\mu_{a(1057)},g_{1057},{\vec{r}}_{i}\right)}\\ & \approx & \frac{\eta_{1057}}{\eta_{1057}^{\prime }}\cdot \frac{{\bar{A}}_{\left(808\right)}}{{{\bar{A}}^{\prime }}_{\left(808\right)}}\propto \frac{n_{\mathrm{QDs}}}{n_{\mathrm{NCs}}} \end{array}$$

where *I*_i_ represents the laser beam intensity reaching on the surface of hybrid nanocomposite particle i; *A*_i(808)_ $A_{i(808)}^{\prime }$ is the dimensionless absorption strength of QDs (NCs) in hybrid nanocomposite particle i, and ${\bar{A}}_{\left(808\right)}$ (${\bar{A}}_{\left(808\right)}^{\prime }$) is the average absorption strength of QDs (NCs) in hybrid nanocomposites; *η*_1057_ ($\eta_{1057}^{\prime }$) is the absolute quantum yield of emission at 1057 nm of QDs (NCs) in hybrid nanocomposites; *φ*_i_ is the signal response of the experimental system for the emission (1057 nm) from particle i; *g*_1057_ is scattering anisotropic factor for light at 1057 nm; ${\vec{r}}_{i}$ is the position vector between the particle i and the slit of spectrometer of the experimental system. According to Equation (2), *I*_QDs‐1057_(808)/*I*_NCs‐1057_(808) is ultimately proportional to the average molar ratio of QDs to NCs (*n*_QDs_/*n*_NCs_) in hybrid nanocomposites.

##### Estimation of *n*_QDs_/*n*_NCs_

The average molar ratio of QDs to NCs in hybrid nanocomposites was calculated to be ≈3:1 by the following equation

(3) $$\frac{n_{\mathrm{QDs}}}{n_{\mathrm{NCs}}}\approx \frac{I_{\mathrm{QDs}}\left(808\right)}{I_{\mathrm{NCs}}\left(808\right)}\cdot \frac{\sigma_{\mathrm{Nd}}\cdot V\cdot N\cdot c\cdot QY_{\mathrm{NCs}}}{\sigma_{\mathrm{QDs}}\cdot QY_{\mathrm{QDs}}}$$

where the ratio of integrated PL intensity of QDs to that of NCs under 808 nm excitation (*I*_QDs_(808)/*I*_NCs_(808)) is 3.35; *σ*_QDs_ (≈0.5 × 10^−16^ cm^2^) and *σ*_Nd_ (≈0.4 × 10^−19^ cm^2^) are the absorption cross sections of single QD and Nd^3+^ ion, respectively; QY_QDs_ (≈21%) and QY_NCs_ (≈41%) are NIR fluorescence quantum yields of QDs and NCs, respectively; *V* (≈8181.3 nm^3^) is the average volume of core NCs; *N* (≈14.4 nm^−3^) is the molar density of rare earth ions in hexagonal NaLuF_4_ matrix; *c* (5%) is the doping molar concentration of Nd^3+^ ions.

##### Calculation of Thermal Resolution of the Hybrid Nanocomposites Thermometers

The temperature resolution Δ*T* can be given by

(4a) $$\Delta T=\frac{\Delta R}{dR/dT}=\frac{\Delta R}{S_{r}\cdot R}$$

where *S*_r_ (1.5% K^−1^) is the relative thermal sensitivity; Δ*R* is the uncertainty in the determination of *R* (*I*_QDs‐1057_(808)/*I*_NCs‐1057_(808)). Here, Δ*R*/*R* is given by

(4b) $$\begin{array}{ccc} \frac{\Delta R}{R} & \approx & \frac{I_{\mathrm{NCs}}}{I_{\mathrm{QDs}}}\sqrt{{\left(\frac{1}{I_{\mathrm{NCs}}}\right)}^{2}\cdot {\left(\Delta I_{\mathrm{QDs}}\right)}^{2}+{\left(\frac{I_{\mathrm{QDs}}}{{I_{\mathrm{NCs}}}^{2}}\right)}^{2}\cdot {\left(\Delta I_{\mathrm{QDs}}\right)}^{2}}\\ & = & \sqrt{{\left(\frac{\Delta I_{\mathrm{QDs}}}{I_{\mathrm{QDs}}}\right)}^{2}+{\left(\frac{\Delta I_{\mathrm{NCs}}}{I_{\mathrm{NCs}}}\right)}^{2}} \end{array}$$

where Δ*I*_QDs_/*I*_QDs_ ≈ Δ*I*_NCs_/*I*_NCs_ ≈ 2% (the estimated laser instability). Therefore, the thermal resolution was calculated to be about 1.8 °C at 35.0 °C. Note that the thermal resolution of the hybrid nanocomposites thermometers strongly depends on the stability of laser beam. Therefore, better time stability of the excitation source will further improve the thermal resolution.

## Conflict of Interest

The authors declare no conflict of interest.

## Supporting information

Supporting Information

## References

[1] a) Z. Wang , X. W. He , T. Y. Yong , Y. Miao , C. Zhang , B. Z. Tang , J. Am. Chem. Soc. 2020, 142, 512; 31829626 10.1021/jacs.9b11544

[2] a) E. C. Ximendes , W. Q. Santos , U. Rocha , U. K. Kagola , F. Sanz‐Rodríguez , N. Fernández , A. d. S. Gouveia‐Neto , D. Bravo , A. M. Domingo , B. del Rosal , C. D. S. Brites , L. D. Carlos , D. Jaque , C. Jacinto , Nano Lett. 2016, 16, 1695; 26845418 10.1021/acs.nanolett.5b04611

[3] a) F. Vetrone , R. Naccache , A. Zamarrón , A. J. d. l. Fuente , F. Sanz‐Rodríguez , L. M. Maestro , E. M. Rodriguez , D. Jaque , J. G. Solé , J. A. Capobianco , ACS Nano 2010, 4, 3254; 20441184 10.1021/nn100244a

[4] a) X. L. Huang , J. B. Song , B. C. Yung , X. H. Huang , Y. H. Xiong , X. Y. Chen , Chem. Soc. Rev. 2018, 47, 2873; 29568836 10.1039/C7CS00612H PMC5926823

[5] a) P. Taroni , A. Pifferi , A. Torricelli , D. Comelli , R. Cubeddu , Photochem. Photobiol. Sci. 2003, 2, 124; 12664972 10.1039/b209651j

[6] M. Quintanilla , Y. Zhang , L. M. Liz‐Marzán , Chem. Mater. 2018, 30, 2819.

[7] E. N. Cerón , D. H. Ortgies , B. del Rosal , F. Ren , A. Benayas , F. Vetrone , D. Ma , F. Sanz‐Rodríguez , J. G. Solé , D. Jaque , E. M. Rodríguez , Adv. Mater. 2015, 27, 4781. 26174612 10.1002/adma.201501014

[8] a) S. L. Jacques , Phys. Med. Biol. 2013, 58, R37; 23666068 10.1088/0031-9155/58/11/R37

[9] L. Labrador‐Páez , M. Pedroni , A. Speghini , J. Garcia‐Solé , P. Haro‐González , D. Jaque , Nanoscale 2018, 10, 22319. 30468230 10.1039/c8nr07566b

[10] Y. Shen , J. Lifante , N. Fernández , D. Jaque , E. Ximendes , ACS Nano 2020, 14, 4122. 32227917 10.1021/acsnano.9b08824

[11] M. H. Niemz , Laser‐Tissue Interactions, Springer, Berlin Heidelberg 2003.

[12] a) S. Jeong , J. Song , W. Lee , Y. M. Ryu , Y. Jung , S. Y. Kim , K. Kim , S. C. Hong , S. J. Myung , S. Kim , Nano Lett. 2017, 17, 1378; 28125238 10.1021/acs.nanolett.6b04261

[13] F. Bai , D. S. Wang , Z. Y. Huo , W. Chen , L. P. Liu , X. Liang , C. Chen , X. Wang , Q. Peng , Y. D. Li , Angew. Chem. Int. Ed. 2007, 46, 6650. 10.1002/anie.200701355 17661295

[14] a) Y. Wang , G. Liu , L. Sun , J. Xiao , J. Zhou , C. Yan , ACS Nano 2013, 7, 7200; 23869772 10.1021/nn402601d

[15] a) Y. Zhao , C. Riemersma , F. Pietra , R. Koole , C. d. M. Donega , A. Meijerink , ACS Nano 2012, 6, 9058; 22978378 10.1021/nn303217q

[16] a) B. del Rosal , D. Ruiz , I. Chaves‐Coira , B. H. Juárez , L. Monge , G. S. Hong , N. Fernández , D. Jaque , Adv. Funct. Mater. 2018, 28, 1806088;

[17] a) M. Xu , X. M. Zou , Q. Q. Su , W. Yuan , C. Cao , Q. H. Wang , X. J. Zhu , W. Feng , F. Y. Li , Nat. Commun. 2018, 9, 2698; 30002372 10.1038/s41467-018-05160-1 PMC6043590

[18] a) M. Saba , M. Cadelano , D. Marongiu , F. Chen , V. Sarritzu , N. Sestu , C. Figus , M. Aresti , R. Piras , A. G. Lehmann , C. Cannas , A. Musinu , F. Quochi , A. Mura , G. Bongiovanni , Nat. Commun. 2014, 5, 5049; 25266869 10.1038/ncomms6049

[19] G. Itskos , A. Othonos , T. Rauch , S. F. Tedde , O. Hayden , M. V. Kovalenko , W. Heiss , S. A. Choulis , Adv. Energy Mater. 2011, 1, 802.

[20] a) M. Kamimura , T. Matsumoto , S. Suyari , M. Umezawa , K. Soga , J. Mater. Chem. B 2017, 5, 1917; 32263945 10.1039/c7tb00070g

[21] a) Y. Zhong , G. Tian , Z. Gu , Y. Yang , L. Gu , Y. Zhao , Y. Ma , J. Yao , Adv. Mater. 2014, 26, 2831; 24338994 10.1002/adma.201304903

[22] a) G. S. Hong , S. Diao , J. L. Chang , A. L. Antaris , C. X. Chen , B. Zhang , S. Zhao , D. N. Atochin , P. L. Huang , K. I. Andreasson , C. J. Kuo , H. J. Dai , Nat. Photonics 2014, 8, 723; 27642366 10.1038/nphoton.2014.166 PMC5026222

[23] X. J. Zhu , W. Feng , J. Chang , Y. W. Tan , J. C. Li , M. Chen , Y. Sun , F. Y. Li , Nat. Commun. 2016, 7, 10437. 26842674 10.1038/ncomms10437 PMC4742858

[24] R. Z. Wu , J. J. Zhou , L. Lei , S. J. Zhang , Z. Xiao , J. J. Zhang , S. Q. Xu , Chem. Phys. Lett. 2017, 667, 206.

[25] A. F. Pereira , J. F. Silva , A. S. Gouveia‐Neto , C. Jacinto , Sensor Actuat. B Chem. 2017, 238, 525.

[26] L. Marciniak , A. Bednarkiewicz , M. Stefanski , R. Tomala , D. Hreniak , W. Strek , Phys. Chem. Chem. Phys. 2015, 17, 24315. 26327196 10.1039/c5cp03861h

[27] A. Skripka , A. Benayas , R. Marin , P. Canton , E. Hemmer , F. Vetrone , Nanoscale 2017, 9, 3079. 28252155 10.1039/c6nr08472a

[28] E. Carrasco , B. del Rosal , F. Sanz‐Rodríguez , Á. J. de la Fuente , P. H. Gonzalez , U. Rocha , K. U. Kumar , C. Jacinto , J. G. Solé , D. Jaque , Adv. Funct. Mater. 2015, 25, 615.

[29] A. Benayas , B. del Rosal , A. Pérez‐Delgado , K. Santacruz‐Gómez , D. Jaque , G. A. Hirata , F. Vetrone , Adv. Opt. Mater. 2015, 3, 687.

[30] U. Rocha , J. Hu , E. M. Rodriguez , A. S. Vanetsev , M. Rahn , V. Sammelselg , Y. V. Orlovskii , J. G. Sole , D. Jaque , D. H. Ortgies , Small 2016, 12, 5394. 27552716 10.1002/smll.201600866

[31] D. Wawrzynczyk , A. Bednarkiewicz , M. Nyk , W. Strek , M. Samoc , Nanoscale 2012, 4, 6959. 23072978 10.1039/c2nr32203j

