# Supplementary material for: A Dual‐Excitation Decoding Strategy Based on NIR Hybrid Nanocomposites for High‐Accuracy Thermal Sensing
Source: Adv Sci (Weinh). 2020 Aug 25;7(20):2001589. doi: 10.1002/advs.202001589 (PMC7578878; doi:10.1002/advs.202001589)
Supplement: Supplementary file 1 — Supporting Information [file ADVS-7-2001589-s001.pdf]

© 2020 Wiley-VCH GmbH

ADVANCED SCIENCE
Open Access

Supporting Information

for *Adv. Sci.*, DOI: 10.1002/advs.202001589

A Dual-Excitation Decoding Strategy Based on NIR Hybrid Nanocomposites for High-Accuracy Thermal Sensing

Shaohua Yu, Jin Xu, Xiaoying Shang, Wei Zheng, Ping Huang, Renfu Li, Datao Tu, and Xueyuan Chen**

Supporting Information

A Dual-Excitation Decoding Strategy Based on NIR Hybrid Nanocomposites for High-Accuracy Thermal Sensing

Shaohua Yu, ‡ Jin Xu, ‡ Xiaoying Shang, Wei Zheng, Ping Huang, Renfu Li, Datao Tu,* and Xueyuan Chen*

Supplementary Figures

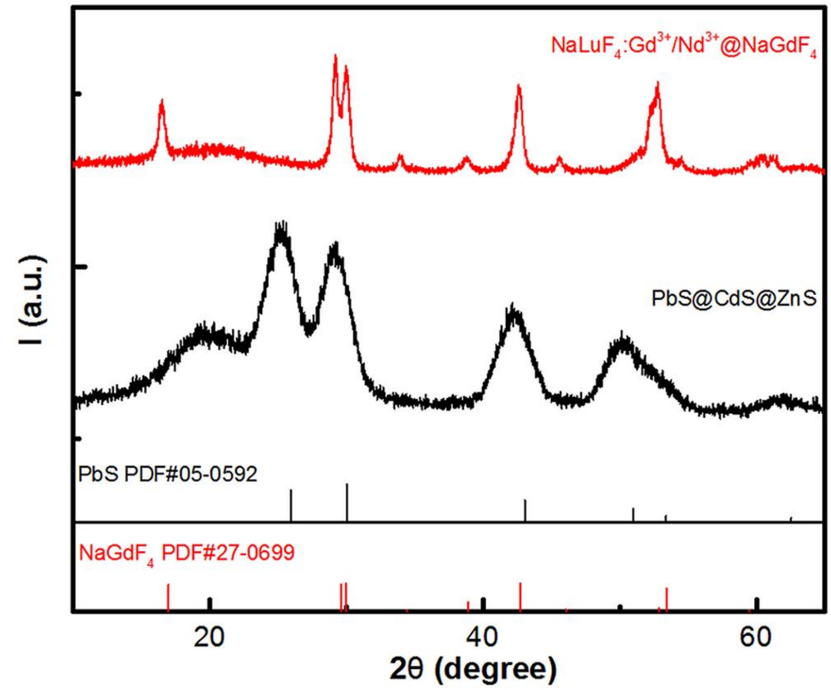

Figure S1. XRD patterns of $NaLuF_4:Gd^{3+}/Nd^{3+}@NaGdF_4$ nanocrystals (NCs) and PbS@CdS@ZnS quantum dots (QDs). All diffraction peaks match well with the standard patterns of hexagonal phase of $NaGdF_4$ (PDF#27-0699) and cubic phase of PbS (PDF#05-0592), respectively.

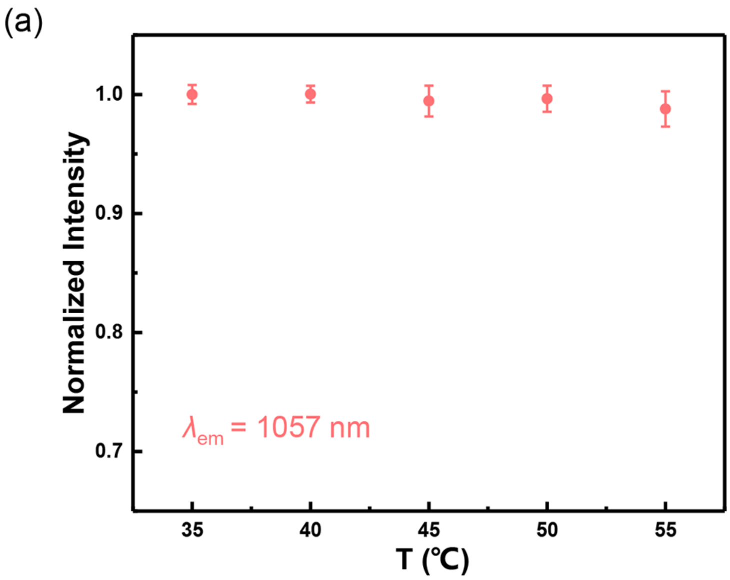

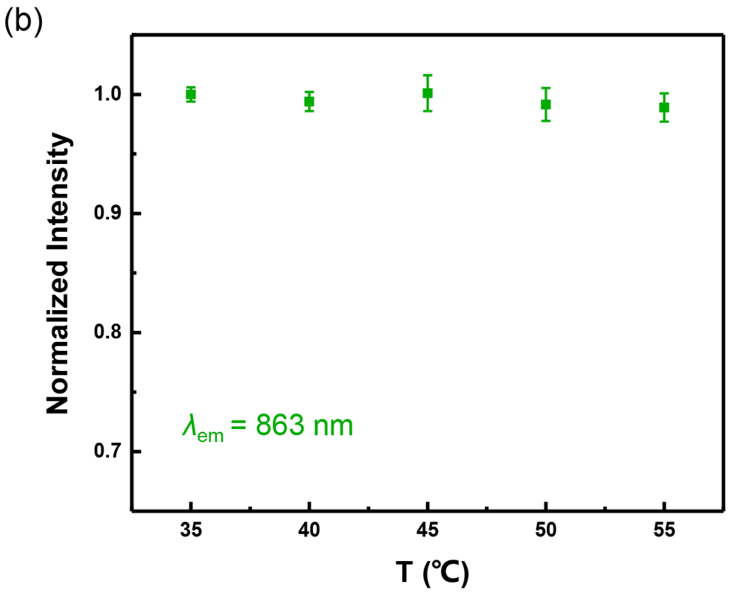

Figure S2. Normalized photoluminescence (PL) intensity at 1057 (a) and 863 nm (b) corresponding to $^4F_{3/2}\rightarrow{}^4I_{11/2}$ and $^4F_{3/2}\rightarrow{}^4I_{9/2}$ transitions of Nd^{3+} as a function of temperature, respectively. It can be seen that the $^4F_{3/2}\rightarrow{}^4I_{11/2}/{}^4I_{9/2}$ emissions of Nd^{3+} remain virtually unaffected by the temperature changes in the 35-55 °C range. Data were presented as average ± standard deviation from three independent measurements.

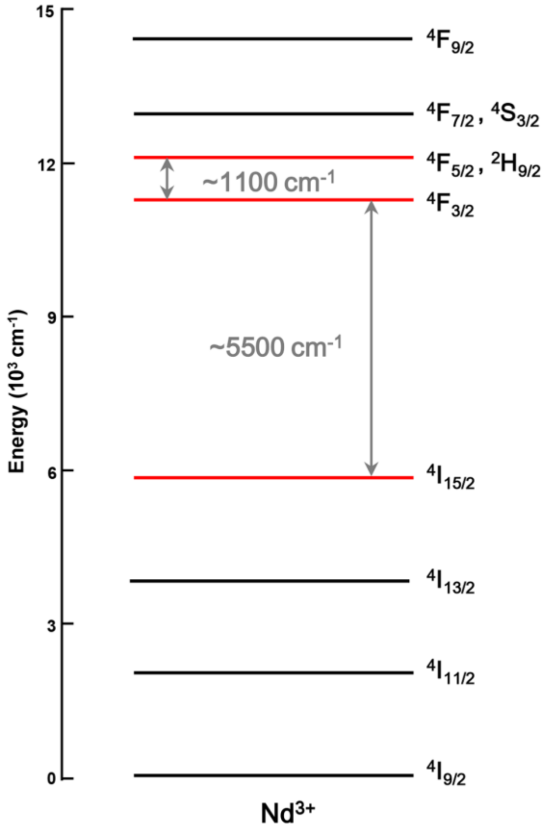

Figure S3. Energy diagram of Nd^{3+} ions. The energy gap between $^4F_{3/2}$ and its nearest lower level $^4I_{15/2}$ is as large as ~ 5500 cm^{-1}, thus the multiphonon relaxation process from $^4F_{3/2}$ to $^4I_{15/2}$ level is negligible. Note that the thermal quenching coefficient for the $^4F_{3/2}$ level itself due to thermal population to its upper $^4F_{5/2}$ lever can be approximately estimated to be $1/[A \cdot \exp(-E_a/K_b T) + 1]$ using the Arrhenius thermal quenching model, where A is constant, E_a is the energy gap between $^4F_{3/2}$ and $^4F_{5/2}$, K_b is Boltzmann constant. Here, the E_a is ~ 1100 cm^{-1} (*i.e.* ~ 137 meV), which is much larger than thermal activation energy of at room temperature (~ 25 meV). As such, the PL intensity of $^4F_{3/2} \rightarrow ^4I_{11/2}/^4I_{9/2}$ transitions of Nd^{3+} in fluoride NCs remains virtually unaffected by the temperature changes from 35 to 55 °C.

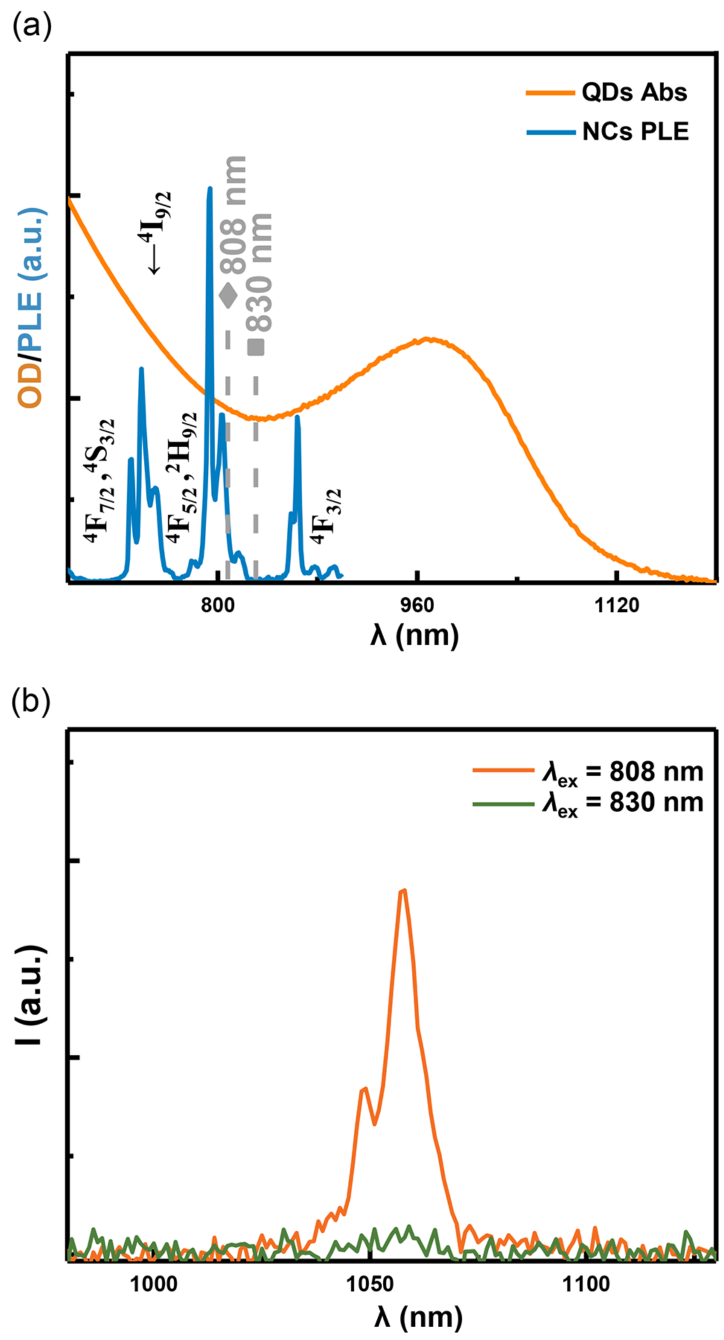

Figure S4. (a) Absorption spectrum of QDs (orange line) and PL excitation (PLE) spectrum of Nd^{3+} doped NCs (blue line). (b) PL emission spectra of NCs under excitations at 808 (orange line) and 830 nm (green line), respectively.

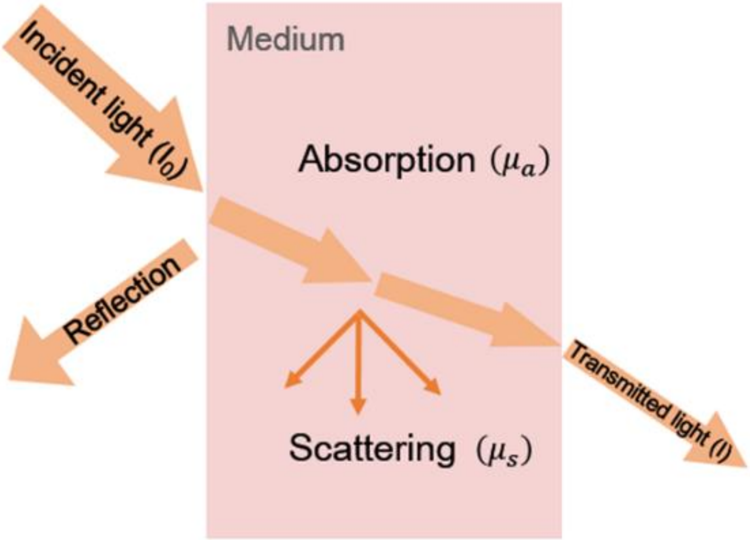

Figure S5. A schematic diagram of light-matter interaction including reflection, refraction, absorption and scattering. Under the diffusion approximation,[1] the intensity at a distance z from the tissue surface could be given in the form: $I = A \cdot \exp(\mu_t z) + B \cdot \exp(\mu_{eff} z)$ with $A + B = I_0$, where $\mu_t = \mu_a + \mu_s$ and $\mu_{eff} = \sqrt{3\mu_a[\mu_a + \mu_s(1 - g)]}$, g is the scattering anisotropic factor, μ_a is the absorption coefficient and μ_s is the scattering coefficient.

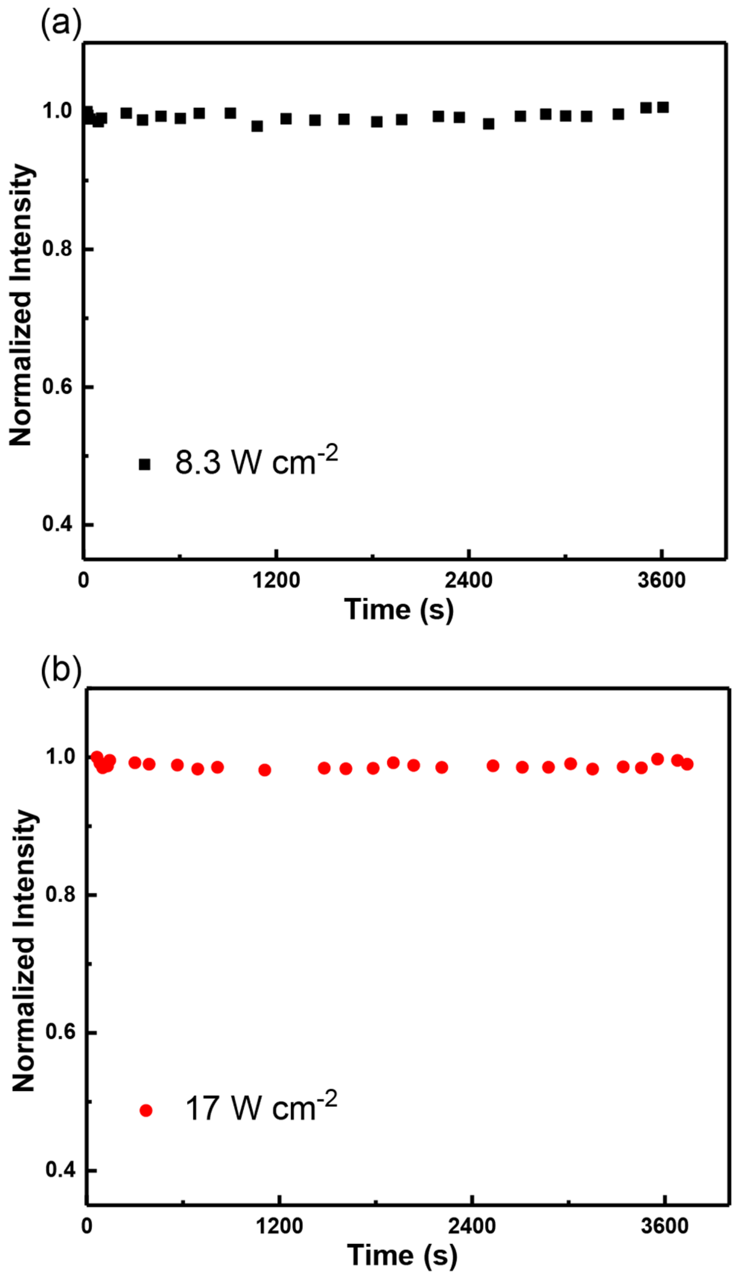

Figure S6. PL intensity versus time for the hybrid nanocomposites dispersion in water (~ 5 mg mL^{-1}) under 808-nm excitation with a power density of ~ 8.3 (a) and ~ 17 W cm^{-2} (b), respectively, which demonstrated the good stability of the hybrid nanocomposites thermometers.

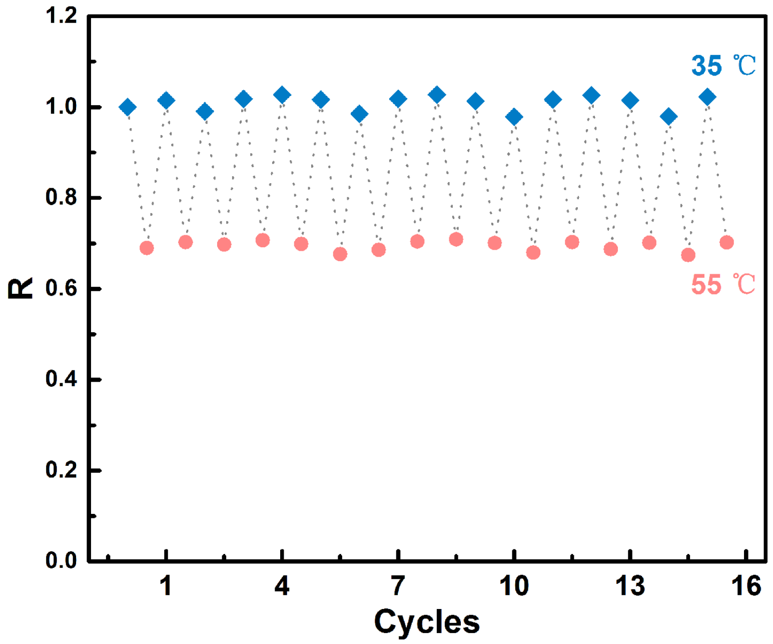

Figure S7. Reversibility of the hybrid nanocomposites thermometers over a span of 16 cycles of heating (55 °C) and cooling (35 °C) processes. This indicates that the optical properties of hybrid nanocomposites are fully reversible without any observable thermal hysteresis in the temperature range of 35-55 °C.

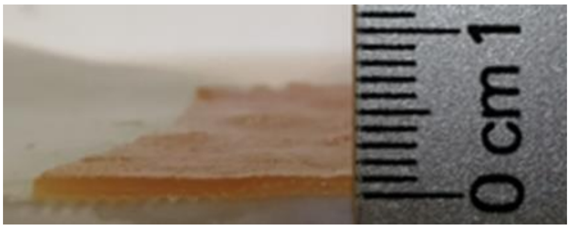

Figure S8. Photograph of pork tissue for the *ex vivo* experiments.

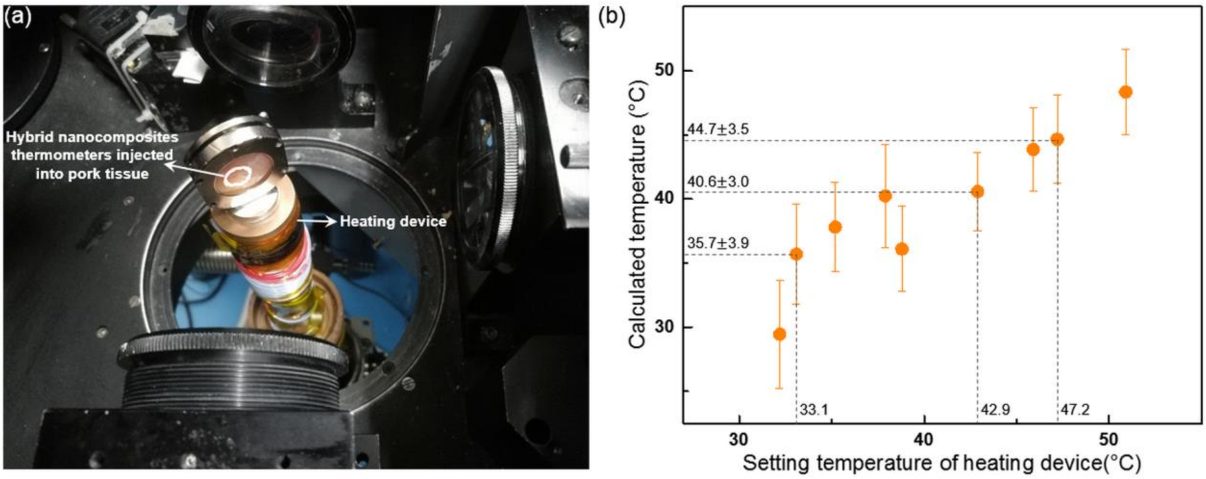

Figure S9. (a) Photograph of experimental setup for the *ex vivo* experiment consisting of direct injection of hybrid nanocomposites thermometers into pork tissue. (b) Calculated local temperatures in pork tissue that was placed on a heating device dynamically set at different temperatures in the range of 30-52 °C. Data were presented as average ± standard deviation from three independent measurements.

Reference

[1] M. H.Niemz, Laser-Tissue Interactions, Springer, Berlin Heidelberg New York, 2003.
